# Bioinformatic identification of potential biomarkers and therapeutic targets in carotid atherosclerosis and vascular dementia

**DOI:** 10.3389/fneur.2022.1091453

**Published:** 2023-01-10

**Authors:** Dongshi Li, Zhixin Huang, Yingyi Dai, Linling Guo, Songbin Lin, Xintong Liu

**Affiliations:** Department of Neurology, Guangdong Second Provincial General Hospital, Guangzhou, Guangdong, China

**Keywords:** biomarkers, DEGs, miRNAs, carotid atherosclerosis, vascular dementia

## Abstract

**Background:**

Vascular disease is the second most common cause of dementia. The prevalence of vascular dementia (VaD) has increased over the past decade. However, there are no licensed treatments for this disease. Carotid atherosclerosis (CAS) is highly prevalent and is the main cause of ischemic stroke and VaD. We studied co-expressed genes to understand the relationships between CAS and VaD and further reveal the potential biomarkers and therapeutic targets of CAS and VaD.

**Methods:**

CAS and VaD differentially expressed genes (DEGs) were identified through bioinformatic analysis Gene Expression Omnibus (GEO) datasets GSE43292 and GSE122063, respectively. Furthermore, a variety of target prediction methods and network analysis approaches were used to assess the protein–protein interaction (PPI) networks, the Gene Ontology (GO) terms, and the pathway enrichment for DEGs, and the top 7 hub genes, coupled with corresponding predicted miRNAs involved in CAS and VaD, were assessed as well.

**Result:**

A total of 60 upregulated DEGs and 159 downregulated DEGs were identified, of which the top 7 hub genes with a high degree of connectivity were selected. Overexpression of these hub genes was associated with CAS and VaD. Finally, the top 7 hub genes were coupled with corresponding predicted miRNAs. hsa-miR-567 and hsa-miR-4652-5p may be significantly associated with CAS and VaD.

## 1. Introduction

Carotid atherosclerosis (CAS) is the manifestation of systemic atherosclerosis (AS) in the carotid arteries, which is currently considered to be closely related to the occurrence of ischemic stroke in the elderly (1, 2). Contrary to the steady or declining trend of most diseases, the prevalence of AS has been proven to be increased in both men and women (3). Vascular dementia (VaD) has existed as a health problem for many years and is widely considered the second most common cause of cognitive impairment following Alzheimer's disease (AD) (4). VaD may be secondary to systemic or local impacts of vascular disease. Common risk factors, such as smoking, hypertension, and diabetes mellitus, can increase the risk of developing coronary artery disease, as well as dementia and cerebrovascular disease (5). Previous studies demonstrated that cerebrovascular disease was a major cause of cognitive impairment or dementia in patients without a clear history of stroke (6). CAS, as one of the vascular contributions to cognitive impairment and dementia, has provoked multitudinous debates and investigations recently, based on epidemiology and preclinical, neuropathology, physiological, and neuroimaging research. Research in recent years has highlighted the role of CAS, which not only is a major cause of cognitive impairment but also provides additional support for other factors that contribute to dementia, including neurodegenerative diseases such as AD (7). In recent years, researchers have become increasingly interested in the treatment of CAS and VaD. In particular, immunotherapy and targeted therapy have improved the therapeutic outlook. However, the efficiency of treatment is defective.

In this study, we identified co-expressed differentially expressed genes (co-DEGs) of persistent CAS and VaD. The screened DEGs were also used for Gene Ontology (GO) functional annotation analysis and the Kyoto Encyclopedia of Genes and Genomes (KEGG) pathway enrichment analysis. Then, we established a protein–protein interaction (PPI) network using the Search Tool for the Retrieval of Interacting Genes (STRING) database and visualized it using Cytoscape software to identify CAS- and VAD-related hub genes. Finally, we predicted microRNAs (miRNAs) in patients with VaD-sensitive CAS.

## 2. Materials and methods

### 2.1. Data source

In this study, the gene expression dataset was analyzed from the NCBI GEO database (https://www.ncbi.nlm.nih.gov/geo/). The GSE43292 dataset based on the GPL6244 platform ([HuGene-1_0-st] Affymetrix Human Gene 1.0 ST Array) included 32 atheroma plaque and 32 macroscopically intact tissue (control samples) from 32 patients with hypertension. The GSE122063 dataset was based on the GPL16699 platforms (Agilent-039494 SurePrint G3 Human GE v2 8x60K Microarray 039381), and the data of the frontal and temporal cortex tissue included 4 VaD samples and 11 non-demented controls (Control). All data were freely available online. This study did not involve any experiments performed on humans or animals by any of the authors.

### 2.2. Screening for DEGs

To verify the shared and unique genes in CAS and VaD, we conducted the DEG analysis on additional CAS and VaD datasets (GSE43292 and GSE122063). The DEGs were detected using the GEO2R online analysis tool (https://www.ncbi.nlm.nih.gov/geo/geo2r/), and the adjusted *p*-value and |logFC| were calculated. An adjusted *p*-value of < 0.05 and |logFC|≥0.5 cutoff criteria were used to identify genes as DEGs. Each dataset underwent statistical analysis, and the intersecting part was identified using the Venn diagram webtool (bioinformatics.psb.ugent.be/webtools/Venn/).

### 2.3. Functional enrichment analysis of DEGs

A common effective method to reveal functional enrichment is GO analysis (8). Biological process (BP), molecular function (MF), and cellular component (CC) are the three categories under which GO enrichment can be classified. The functional interpretation and practical application of genomic information were related to KEGG enrichment analysis (9). In this study, CAS- and VaD-DEGs were subjected to GO annotation analysis and KEGG pathway enrichment analysis utilizing bioinformatics tools from the Database for Annotation, Visualization, and Integrated Discovery (DAVID Gene Functional Classification Tool, https://david.ncifcrf.gov/) and REACTOME databases (http://www.reactome.org) (10). GO terms and KEGG maps of biological functions were associated with a p-value of < 0.01, and gene counts of >10 were considered to be significantly enriched.

### 2.4. Protein–protein interaction (PPI) network construction and hub gene identification

The STRING database (http://string-db.org/) is designed to predict protein functional associations and analyze the PPI information. The STRING database was mapped to the previously discovered DEGs to assess the potential PPI relationship. PPIs of DEGs with a score of >0.4 were selected. Then, the PPI network was constructed and visualized using Cytoscape software (www.cytoscape.org/). The stability of the entire network was more dependent on the higher-degree nodes. The Cytoscape cytoHubba plug-in was used to select the hub genes. The top 7 genes in our analysis were selected as hub genes.

### 2.5. Identification of hub genes associated with nervous diseases

To generate expanded networks and predict novel associations, the comparative toxicogenomics database (CTD, http://ctdbase.org/) was applied to identify integrated chemical–gene, chemical–disease, and gene–disease interactions (11). These data were utilized to analyze the relationship between gene products and nervous system disorders. In this study, the relationships between hub genes and nervous system disorders and the association or an implied association were identified.

### 2.6. microRNA target prediction

To predict potential microRNA targeting, we used online prediction tools utilizing the microRNA Data Integration Portal (mirDIP) (http://ophid.utoronto.ca/mirDIP) (12), miRDB (http://mirdb.org/) (12), and TargetScan (http://www.targetscan.org/vert_71/) (13). Subsequently, we predicted which of the selected miRNAs could target hub genes using the mirDIP, miRDB, and TargetScan software. We determined candidate miRNAs based on higher predicted scores for ≥2 prediction tools for each hub gene. The intersecting miRNAs of each hub gene were identified using the Venn diagram webtool (bioinformatics.psb.ugent.be/webtools/Venn/). The target prediction of miRNA with more than two hub genes expressed was carried to analyse. Finally, to evaluate interactions between hub genes involved in CAS and VaD and miRNAs previously discovered using prediction methods, we used microRNA target prediction with online tools from Diana-miRPath (DIANA TOOLS, V.3, https://dianalab.e-ce.uth.gr/html/mirpathv3/) (14).

## 3. Results

### 3.1. Identification of DEGs associated with the progression of CAS and VaD

**Figure 1:**
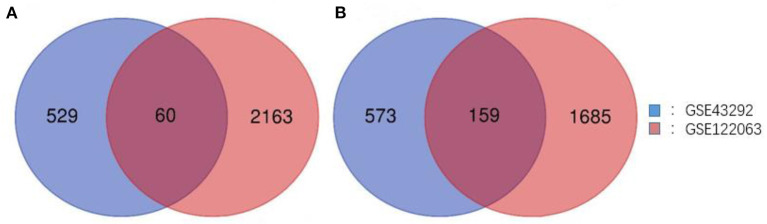
Venn diagram of DEGs common to all two GEO datasets. **(A)** Upregulated genes. **(B)** Downregulated genes.

Two gene expression profiles (GSE43292 and GSE122063) were selected in this study, and CAS- and VaD-DEGs were verified. A total of 1,321 DEGs were identified from GSE43292, including 589 upregulated genes and 732 downregulated genes. From GSE122063, 4,067 DEGs, including 2,223 upregulated genes and 1,844 downregulated genes, were identified. Venn analysis was then used to determine the intersection of the DEG profiles (Figure 1). Finally, 219 DEGs were significantly expressed differently in the two groups, with 60 significantly upregulated genes and 159 considerably downregulated genes.

### 3.2. Functional enrichment analysis in co-DEGs

**Figure 2:**
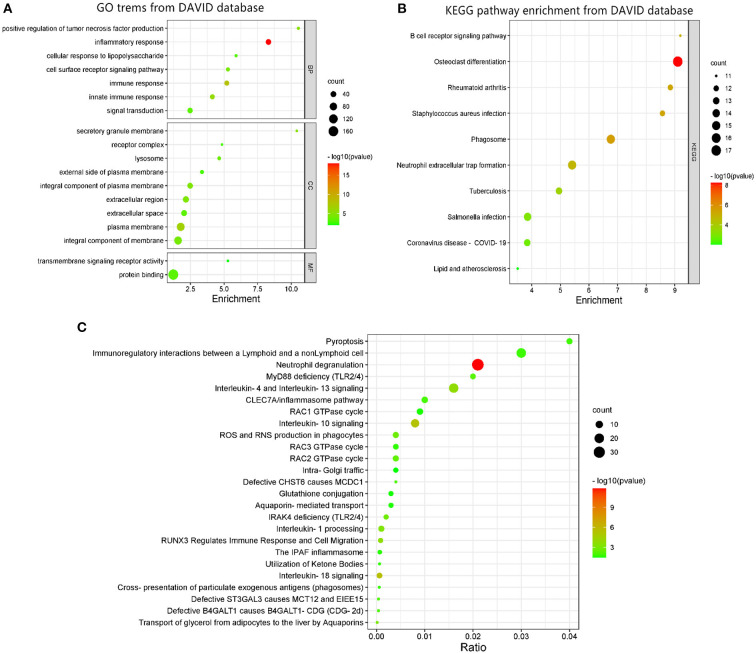
GO terms and KEGG pathway enrichment: **(A)** GO term enrichment for DEGs. **(B)** KEGG pathway of DEGs. **(C)** Functional and pathway enrichment of DEGs from REACTOME database. Dot sizes represent counts of enriched DEGs, and dot colors represent negative Log10-*p*-values.

DAVID was used to perform GO function and KEGG pathway enrichment analysis to comprehend the biological classification of DEGs (Figure 2). GO analysis revealed that the biological processes of DEGs were significantly enriched in inflammatory response (counts = 36, *p* < 0.001), immune response (counts = 27, *p* < 0.001), innate immune response (counts = 26, *p* < 0.001), positive regulation of tumor necrosis factor production (counts = 12, *p* < 0.001), cell surface receptor signaling pathway (counts = 17, *p* < 0.001), signal transduction (counts = 33, *p* < 0.001), and cellular response to lipopolysaccharide (counts = 12, *p* < 0.001). Cell components enriched with DEGs included the plasma membrane (counts = 94, *p* < 0.001), secretory granule membrane (counts = 11, *p* < 0.001), integral component of plasma membrane (counts = 39, *p* < 0.001), extracellular region (counts = 48, *p* < 0.001), integral component of membrane (counts = 89, *p* < 0.001), lysosome (counts = 15, *p* < 0.001), extracellular space (counts = 41, *p* < 0.001), external side of plasma membrane (counts = 16, *p* = 0.0018), and receptor complex (counts = 11, *p* = 0.0018). Cell components enriched with DEGs included the plasma membrane (counts = 94, *p* < 0.001), secretory granule membrane (counts = 11, *p* < 0.001), integral component of plasma membrane (counts = 39, *p* < 0.001), extracellular region (counts = 48, *p* < 0.001), integral component of membrane (counts = 89, *p* < 0.001), lysosome (counts = 15, *p* < 0.001), extracellular space (counts = 41, *p* < 0.001), external side of plasma membrane (counts = 16, *p* = 0.0018), and receptor complex (counts = 11, *p* = 0.0018). The molecular functions of DEGs were enriched in protein binding (counts = 169, *p* < 0.001) and transmembrane signaling receptor activity (counts = 11, *p* = 0.009). KEGG pathway analysis revealed that DEGs were mainly enriched in osteoclast differentiation (counts = 17, *p* < 0.001), phagosome (counts = 15, *p* < 0.001), rheumatoid arthritis (counts = 12, *p* < 0.001), *Staphylococcus aureus* infection (counts = 12, *p* < 0.001), B cell receptor signaling pathway (counts = 11, *p* < 0.001), neutrophil extracellular trap formation (counts = 15, *p* < 0.001), tuberculosis (counts = 13, *p* < 0.001), *Salmonella* infection (counts = 14, *p* < 0.001), Coronavirus disease (counts = 13, *p* = 0.0017), and lipid and AS (counts =11, *p* = 0.0099).

GO terms enrichment identified additional associations using the REACTOME database as shown in Figure 2. The most significant pathways included neutrophil degranulation, interleukin-18 signaling, interleukin-10 signaling, interleukin-4, and interleukin-13 signaling, RUNX3 regulates immune response and cell migration, interleukin-1 processing, transport of glycerol from adipocytes to the liver by aquaporins, ROS and RNS production in phagocytes, IRAK4 deficiency (TLR2/4), RAC2 GTPase cycle, MyD88 deficiency (TLR2/4), defective B4GALT1 causes B4GALT1-CDG (CDG-2d), defective ST3GAL3 causes MCT12 and EIEE15, defective CHST6 causes MCDC1, CLEC7A/inflammasome pathway, pyroptosis, immunoregulatory interactions between a lymphoid and a non-lymphoid cell, cross-presentation of particulate exogenous antigens (phagosomes), RAC3 GTPase cycle, utilization of ketone bodies, the IPAF inflammasome, aquaporin-mediated transport, RAC1 GTPase cycle, glutathione conjugation, and intra-Golgi traffic.

### 3.3. PPI network construction and hub gene identification

**Figure 3:**
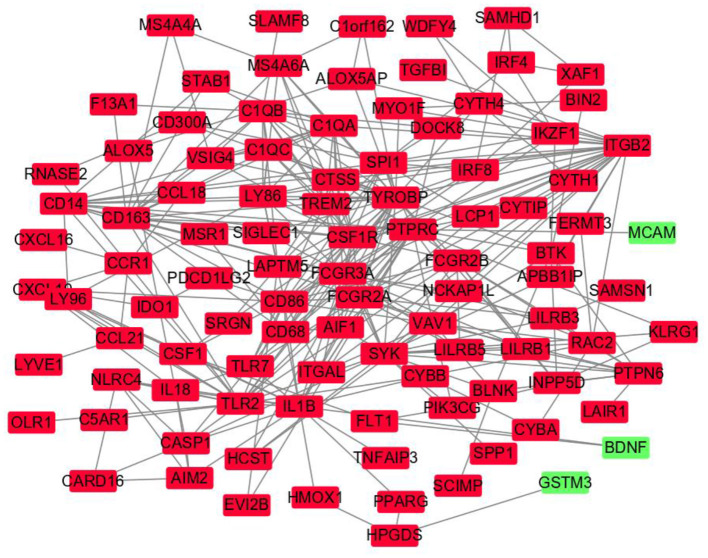
Protein–protein interaction network constructed with the differentially expressed genes. Green nodes represent upregulated genes, and red nodes represent downregulated genes.

The DEGs' protein interactions were predicted using STRING tools. A total of 218 nodes and 1,519 edges were involved in the PPI network, as presented in Figure 3. The top 7 genes based on connectivity degree in the PPI network were identified. The results showed that TYRO protein kinase-binding protein (TYROBP) was the most outstanding gene with connectivity degree = 4,226, followed by colony-stimulating factor 1 receptor (CSF1R; degree = 3,986), baculoviral IAP repeat containing 5 (BIRC5; degree = 60), complement C1q A chain (C1QA; degree = 3,204), complement C1q B chain (C1QB; degree = 2,649), integrin subunit beta 2 (ITGB2; degree = 2751), lymphocyte antigen 86 (LY86; degree = 2,330), and Fc gamma receptor IIIa (FCGR3A; degree = 1,964). All of these hub genes were downregulated in CAS and VaD.

**Figure 4:**
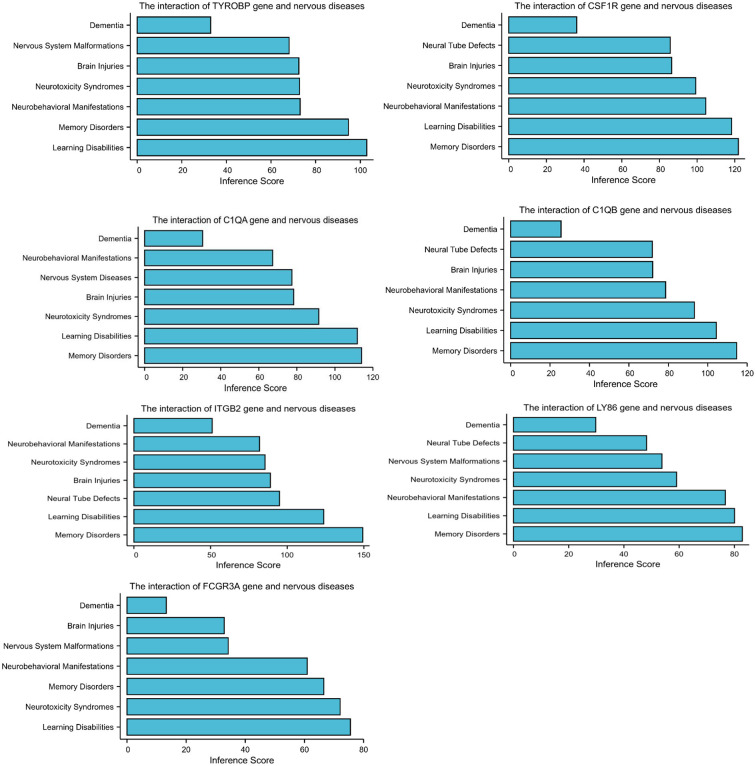
Relationship to nervous system diseases related to co-expressed genes based on the CTD database.

The CTD database showed that the hub genes targeted several nervous system disorders as shown in Figure 4.

### 3.4. Identification of functional and pathway enrichment among predicted miRNAs and co-DEGs

The selected miRNAs targeting each co-DEG involved in CAS and VaD were identified using prediction analysis utilizing mirDIP, miRDB, and TargetScan. The intersecting miRNAs of each hub gene were identified with the Venn diagram webtool as shown in Table 1. We can now comprehend how predicted miRNAs are related to CAS and VaD progress using these data.

**Table 1:** The GO terms and KEGG pathways enrichment among predicted miRNAs and Co-DEGs.

**Predicted miRNAs**	**Genes**	**Category**		***P*-value**
hsa-miR-567	CSF1R FCGR3A	GO terms	Organelle	0.000234
hsa-miR-4652-5p	CSF1R LY86		Cellular nitrogen compound metabolic process	0.002006
			Synaptic transmission	0.011593
			Ion binding	0.02099
			Gene expression	0.023257
		KEGG pathway	Cell adhesion molecules (CAMs)	8.85E-05
			Pancreatic secretion	0.015902
			Calcium signaling pathway	0.015902
			Cardiac muscle contraction	0.029807
			Insulin secretion	0.029807
			Salivary secretion	0.029807
			TGF-beta signaling pathway	0.040109

## 4. Discussion

Carotid atherosclerosis is an independent vascular risk factor for cognitive impairment. Multiple clinical studies support the relationship between AS and VaD, as well as AD. For instance, findings from cerebral autopsies (15) and carotid artery ultrasound (16) examinations show that stenosis occurs more frequently in VaD and AD than in normal cognition and that VaD has a stronger connection with AS than AD with AS. According to other studies, dementia and AS are found to be related to each other (17). Certified biomarkers with better ability to screen, diagnose, and monitor disease were used for precision therapy and prognosis. Regarding this, gene expression, RNA sequencing, and bioinformatic analysis have become increasingly popular and have provided a comprehensive screening of biomarkers as well as a way to explain the fundamental role of biomarkers in the pathology of diseases. Our findings revealed that a number of genes and miRNAs may play roles in the progression of VaD and AD and have the potential to be therapeutic targets. In this study, we exclusively focused on seven hub genes and two miRNAs.

As expected, the biological alterations in the pathogenesis of AS and VaD associated with inflammatory response and immune response were revealed by the GO and KEGG enrichment analyses. AS, inflammatory, and immune responses are known to be related to VaD. A wealth of experimental and clinical evidence demonstrates unequivocally that inflammation and immune response are clearly key players in the pathogenesis of AS and VaD (18–20). An analysis of the production of enzymes and cytokines and cell interactions could provide a better understanding of plaque development. The presence of different immune cells in atherosclerotic plaques illustrates the idea of inflammation. The primary players in the initiation and development of atherosclerotic plaques are monocytes and macrophages. The TH1-associated cytokines interferon- (IFN-), tumor necrosis factor (TNF), interleukin-2 (IL-2), interleukin-3 (IL-3), and lymphotoxin, which can activate T lymphocytes, macrophages, and other plaque components and thus exacerbate the inflammatory response, are expressed by many CD4+ T cells in the plaque (21). The serum levels of C-reactive protein (CRP) and pro-inflammatory cytokines, which serve as the biomarkers for systemic inflammation, are reported to have a significant relationship with vascular injury (22). Leukocytes and inflammatory cells that adhere to microvessels are promoted by the interleukin-1 (IL-1) hormone. Several related pro-inflammatory cytokines are activated by IL-1, which also upregulates leukocyte migration. Vascular reocclusion, thrombosis, and neuronal injury are all made worse in VaD by IL-1-induced activity (23, 24). Increased levels of interleukin-18 (IL-18) have also been considered in relation to cerebrovascular dysfunctions, where apoptosis caspases function as key mediators (25).

A PPI network was used to investigate the regulation mechanism of the DEGs, and seven hub genes were identified, including TYROBP, CSF1R, C1QA, C1QB, ITGB2, LY86, and FCGR3A. All of these genes were downregulated in CAS and VaD. The CTD database revealed these DEGs are clearly associated with learning disabilities and memory disorders.

A tyrosine-based activation motif on the immune signaling adaptor TYROBP, also known as the DNAX-activating protein of 12 kDa (DAP12), encodes a transmembrane signaling polypeptide. This receptor, which transduces immunological signals, is widely expressed in neutrophils, monocytes/macrophages, and natural killer (NK) cells (26). Apolipoprotein E (APOE) mice were found to have high levels of DAP12 expression, which furthered atherosclerotic plaque development through the TREM-1/DAP12 pathway (27). Another vascular transcriptomics study confirmed that, given a high fat/cholesterol (HFC) diet, Tibetan minipig AS models had higher TYROBP mRNA levels (28). The critical role of TYROBP in the pathophysiology and progression of AD has been confirmed in several reports. A postmortem investigation of patients with AD exposed upregulation of TYROBP, indicating that TYROBP is an important regulator of AD processes (29). Additionally, the expression of TYROBP is elevated in AD mouse models, and patients with familial early-onset AD have been discovered to have rare TYROBP missense coding variations (30, 31). However, the role of TYROBP in VaD pathogenesis is still unknown. CSF1R, a member of the class III transmembrane tyrosine kinase receptor family, is a product of the proto-oncogene c-fms. By regulating the biological activity of macrophages *via* the CSF1R, colony-stimulating factor 1 (Csf1), which is secreted by endothelial cells, advances AS (32). AS has been shown to progress more slowly when CSF1R is suppressed because it prevents macrophage proliferation (33). Small-molecule inhibitors targeting CSF1R were used to eliminate the microglia. These inhibitors have been shown to modify the pathological processes of some neurodegenerative disorders, such as AD, frontotemporal dementia, Parkinson's disease, multiple sclerosis, and other neurodegenerative diseases. The fundamental roles of CSF1R in brain development are indicated by the global deficits in brain development shown in the homozygous Csf1r knockout (Csf1r-/-) mice models, including lateral ventricle extension, olfactory bulb atrophy, neocortex thinning, and functional abnormalities of the sensory nervous system (34). The serum complement subcomponent C1q's C-chain or B-chain polypeptides, which are crucial in the regulation of the complement system and have significant effects on neurological diseases, are encoded by the genes C1QA or C1QB. Aged C1QA-knockout mice had lower levels of cognitive and memory deterioration according to reports (35). According to a study, C1QB might be associated with AS (36). Leukocyte adherence and migration into the intima are induced by ITGB2, also known as CD18, a main member of the integrin family. Once in the intima, ITGB2 triggers a number of important inflammatory molecules and immune mediators, including ICAM1 and VCAM1, thus activating the endothelial cells and stimulating the macrophages to absorb modified lipoproteins (37). As a result, it facilitates the development of atherosclerosis. LY86, commonly referred to as protein MD-1, is a secreted glycoprotein connected to RP105. A receptor for the Fc portion of immunoglobulin G is encoded by the gene FCGR3A (38). In AD, the Fc gamma receptor (FCGR) has been shown to exacerbate neurodegeneration. However, studies of LY86 and FCGR3A in CAS and VaD are relatively uncommon.

We found that hsa-miR-567 and hsa-miR-4652-5p were expressed by the co-DEGs and may be potential biomarkers of CAS and VaD. Multiple miRNAs are more abundant in particular organs, tissues, and cell types, such as various regions of the brain or neurons. Mature miRNA are known to play roles in learning and memory, synaptic plasticity, neurogenesis, neuronal differentiation, and neuroprotection (39). Recent studies have demonstrated that miRNAs regulate AS-prone genes and have an impact on post-transcriptional control of gene expression, contributing to the pathophysiology of AS (40). In AS, abnormal regulation of miRNAs promotes the development of VaD as the butterfly effect. The target genes of these selected aberrant miRNAs were involved in a variety of binding processes, including organelle, cellular nitrogen compound metabolic process, synaptic transmission, ion binding, and gene expression, according to the GO analysis. The KEGG enrichment analysis of miRNA indicated the involvement of cell adhesion molecules, pancreatic secretion, the calcium signaling pathway, cardiac muscle contraction, insulin secretion, salivary secretion, and the TGF-beta signaling pathway, which are implicated in the pathogenesis of CAS and VaD. These enriched pathways, such as the TGF-beta signaling pathway, play an important role in CAS and VaD. One of the primary drivers of vascular inflammation associated with atherosclerosis is endothelial TGF-β signaling. Inhibition of endothelial TGF-βsignaling in hyperlipidemic mice reduces vessel wall inflammation and vascular permeability, stops the evolution of the disease, and reverses lesions that have already formed (41). Transgenic mice that constitutively overexpress the cytokine TGF-β1 (TGF mice) exhibit cerebrovascular alterations similar to those in AD as well as vascular cognitive impairment and vascular dementia. According to research, angiotensin II plays a crucial role in TGF-β1-induced cerebrovascular dysfunction and neuroinflammation *via* angiotensin II type-1 receptor (AT1R) -mediated mechanisms (42). Therefore, the correlation of pathogenic processes within miRNA-target genes is implied by these predicted biological processes and signaling pathways. The results need more extensive experimental confirmation, such as the function and interaction assays of important genes *in vitro* and *in vivo*, even if this work described prospective novel biomarkers for CAS and VaD.

## 5. Conclusion

These findings support the value of our study in that it offers a comprehensive understanding of the pathogenesis in relation to gene expression and the severity of CAS and VaD, respectively. The hub genes TYROBP, CSF1R, C1QA, C1QB, ITGB2, LY86, and FCGR3A may be associated with CAS and VaD. The miRNAs, such as hsa-miR-567 and hsa-miR-4652-5p, for each co-DEGs may be potential biomarkers or therapeutic targets for CAS and VaD. The majority of these have not yet been published, and future research initiatives may be developed to confirm the underlying mechanisms through more extensive investigations.

## Data availability statement

The datasets presented in this study can be found in online repositories. The names of the repository/repositories and accession number(s) can be found in the article/supplementary material.

## Ethics statement

Ethical review and approval was not required for the study on human participants in accordance with the local legislation and institutional requirements. Written informed consent from the patients/participants or patients/participants' legal guardian/next of kin was not required to participate in this study in accordance with the national legislation and the institutional requirements.

## Author contributions

DL was responsible for bioinformatics analysis, prepared figures and tables, drafted, and wrote the manuscript. LG and SL carried out data download and preprocessing. XL revised this manuscript and proofread the manuscript and figures. YD and ZH conceived the concept, instructed bioinformatics analysis, supervised results, and were responsible for its financial support. All authors approved the final manuscript.
